# Experimental and theoretical thermal equations of state of MgSiO_3_ post-perovskite at multi-megabar pressures

**DOI:** 10.1038/srep22652

**Published:** 2016-03-07

**Authors:** Takeshi Sakai, Haruhiko Dekura, Naohisa Hirao

**Affiliations:** 1 Geodynamics Research Center, Ehime University, Matsuyama 790-8577, Japan; 2 Japan Synchrotron Radiation Research Institute, Hyogo 679-5198, Japan

## Abstract

The MgSiO_3_ post-perovskite phase is the most abundant silicate phase in a super-Earth’s mantle, although it only exists within the Earth’s lowermost mantle. In this study, we established the thermal equation of state (EoS) of the MgSiO_3_ post-perovskite phase, which were determined by using both laser-heated diamond anvil cell and density-functional theoretical techniques, within a multi-megabar pressure range, corresponding to the conditions of a super-Earth’s mantle. The Keane and AP2 EoS models were adopted for the first time to extract meaningful physical properties. The experimentally determined Grüneisen parameter, which is one of the thermal EoS parameters, and its volume dependence were found to be consistent with their theoretically obtained values. This reduced the previously reported discrepancy observed between experiment and theory. Both the experimental and theoretical EoS were also found to be in very good agreement for volumes at pressures and temperatures of up to 300 GPa and 5000 K, respectively. Our newly developed EoS should be applicable to a super-Earth’s mantle, as well as the Earth’s core-mantle boundary region.

Since 2004, when MgSiO_3_ post-perovskite (PPv) was discovered to be the final phase of the Earth’s lower mantle^1^,^2^, numerous studies have been conducted on its physical properties, phase boundary conditions, and chemical (Fe and Al) effects, in order to understand the seismological features of the D” layer of the Earth^3^. Among these, the equations of state (EoS) of the MgSiO_3_ end member, in particular, provide very fundamental information, i.e., a pressure-volume-temperature (*P-V-T*) relationship. Many studies on the EoS have been done at the *P-T* conditions of the lowermost mantle from *ab initio* computations using density-functional theory (DFT)^2^,^4^,^5^,^6^,^7^, laser-heated diamond anvil cell (LHDAC) experiments^8^,^9^,^10^,^11^, and shock experiments^12^.

There is a volume mismatch between the previously reported EoSs. In the case of the EoS determined by LHDAC, there are two probable reasons for this discrepancy. The first reason is the difference in the pressure scales adopted in the experiments, and the second is the effect of the deviatoric stress within the sample. For example, even if the same pressure scale is used, a small volume difference (approximately 0.3% (0.4 Å^3^) at 120 GPa) would still appear between the EoS reported by Guignot *et al.*^10^ and Komabayashi *et al.*^11^, which is caused by the effect of the uniaxial (deviatoric) stress. Therefore, the effects of this should be taken into consideration when determining the EoS. To the best of our knowledge, only Guignot *et al.*^10^ took this effect into account in their analysis.

For the EoS at high temperature, the Mie-Grüneisen-Debye (MGD) model is often used. Guignot *et al.*^10^ reported the model parameters for high temperature MGD EoS, but they used a Grüneisen parameter (*γ*) determined only by theoretical calculation and not determined by the LHDAC study. Although Mosenfelder *et al.*^12^ measured the *γ* based on their shock experiment and found that their obtained value (γ_0_ = 2.61 ± 0.67) differed from the theoretically predicted one (*γ*_*0*_ = 1.5–1.6) primary due to the limitation of the experimental data. Moreover, they used a conventional form of the volume dependence in *γ*, i.e., *γ* = *γ*_*0*_(*V/V*_*0*_)^*q*^, where *V*_*0*_ and *q* represent the zero pressure volume and the dimensionless parameter, respectively. As a result, the volume dependence derived differed greatly from the theoretical value^5^,^8^ calculated based on the form of the equation detailed by Al’tshuler *et al.*^13^: 
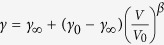
, where 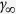 and *β* represent the Grüneisen parameter at infinite pressure and the dimensionless parameter, respectively.

These studies targeted the Earth’s lowermost mantle. The maximum pressure is 136 GPa in the Earth’s mantle. However, recently, one after another, super-Earths, which have masses of a few times that of the Earth, have been found in the extra solar system^14^. If super-Earths have a similar bulk composition with the Earth, MgSiO_3_ PPv is believed to be an abundant silicate phase in the mantles of these huge terrestrial planets^15^. Although the pressure conditions of these super-Earths’ mantles reach several hundreds of GPa, previously reported EoS of PPv, derived by LHDAC experiments, were limited up to approximately 150 GPa, and theoretical calculations were also limited up to 200 GPa. Moreover, PPv is also expected to exist in Uranus’s and Neptune’s rocky cores in which the pressure conditions reach approximately 700–800 GPa. The direct determination of the compression behavior of PPv at multi-megabar pressures is, therefore, important to the understanding of a super-Earth’s deep interior, and Uranus’s and Neptune’s rocky cores.

In this study, we derived the thermal EoS of PPv using primary pressure scales (see Method), with consideration of the effects of uniaxial stress. We extended the experimental pressure up to 265 GPa and the calculations up to a pressure and temperature of 1.2 TPa and 5000 K, respectively. We determined the *γ* and its volume dependence, using the Al’tshuler *et al.*’s form^13^, both experimentally and theoretically. Moreover, the Keane and AP2 EoS models (All the EoS formulae are summarized in Supplementary Information A) were newly adopted to discuss the *P-V-T* relation and its derivatives that extract thermodynamic functions in the multi-megabar pressure range.

## Results and Discussion

### Thermal equation of state for the post-perovskite phase: Fitting the EoS to the experimental data

The results of powder X-ray diffraction experiments and the observed unit cell volumes of PPv phase are summarized in Supplementary Information B. The *P-V-T* data were fitted to the 3BM, Vinet, AP2, and Keane EoS with the MGD model. In the fitting of the experimental data, using the Keane EoS, Slater’s formula for *γ* and the equation of *β* calculated by using *γ*_*0*_ and *γ*_*∞*_ were used to reduce the number of fitting parameters (see Supplementary Information A). In the EoS fitting, using the Keane model (fit 5), we successfully determined all of the parameters based on the present experimental data. The obtained *γ*_*∞*_ and 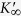 values satisfy the thermodynamic constraints^16^ *γ*_*∞*_ > 2/3 and 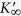 > 5/3. The EoS parameters are summarized in Table 1 with the results of previous studies. The root-mean-square (RMS) of the fitting for volume and pressure were approximately 0.1 Å^3^ and 0.8 GPa, respectively, for the experimental data (fits 5–7). However, the errors in the parameters are quite large for fit 5. These errors yield an unrealistically large volume (or pressure) error compared to the observed data. Alternatively, if we fix the *V*_*0*_ and 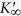 (and *γ*_*∞*_) the errors become reasonably small, and it yields a comparative pressure and volume error to the RMS value (fit 6): 1*σ*_*P*_ = 0.8 GPa and 1*σ*_*V*_ = 0.11 Å^3^ at 100 GPa and 300 K, respectively.

**Figure 1:**
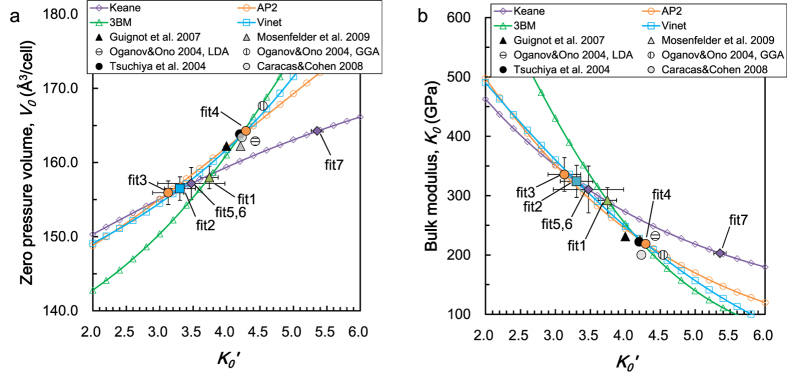
The trade-off relations of (**a**) the zero pressure volume (*V*_*0*_) and (**b**) the bulk modulus (*K*_*0*_) as a function of the derivative *K*_*0*_′. The curves with the diamonds, circles, triangles, and squares show the result of fittings using the Keane, AP2, 3BM, and Vinet EoS models, respectively. The *V*_*0*_ was obtained as a free parameter in fits 1–3 and fits 5–6. In fits 4 and 7, the zero pressure volumes, determined by the *g*-*G* plot, were used. The 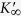 was fixed for the Keane model.

In fits 1–3 and 5 the *V*_*0*_ was relatively small compared to those of the previous reports. Coincidentally, the bulk modulus (*K*_*0*_) was large and its pressure derivative (*K*_*0*_′) was small. It is well known that there is a trade-off relation between these parameters. The trade-off relations of *V*_*0*_ and *K*_*0*_ as a function of *K*_*0*_′ are shown in Fig. 1a,b. The smaller *V*_*0*_ and larger *K*_*0*_ were obtained when *K*_*0*_′ is small. This tendency has a large gradient, and thus it leads to fact that these parameters easily scatter, whereas *γ* was maintained almost constantly at approximately 1.5 in all of the fittings. Although both our and previous results are on the trade-off lines, the determination of the absolute value is the difficult problem.

In particular, the determination of the zero pressure volume is a difficult problem for the high pressure phases, such as the PPv phase, because these phases are not thermodynamically stable at ambient pressures. In these cases, the *g-G* plot^17^ was used for the determination of *V*_*0*_^18^,^19^, where *g* and *G* are the strain parameter and the normalized stress, respectively. The *G* includes a pressure term, and hence, its result depends on which pressure scale is applied. As a consequence, the *g-G* plot yielded a *V*_*0*_ of 161.01 ± 0.38 Å^3^ for the 3BM, 165.64 ± 0.45 Å^3^ for the Vinet, 163.45 ± 0.42 Å^3^ for the AP2, and 164.26 ± 0.42 Å^3^ for the Keane model. Conversely, our theoretical calculations, from −10 to 1200 GPa, enabled us to determine *V*_*0*_ without any extrapolation. The theoretical data yields a *V*_*0*_ of 164.22 ± 0.06 Å. The experiment and the calculation were done completely independently, and it was found that the experimental *V*_*0*_ value derived using the Keane model and the theoretical *V*_*0*_ value were remarkably consistent. The AP2 model also yielded similar *V*_*0*_ values, and these values are consistent within the error of the result reported by Tsuchiya *et al.*^4^, whereas the 3BM and Vinet models yielded very different values. In fit 7, the *V*_*0*_ was fixed to be 164.26 Å^3^, and *γ*_*∞*_ and 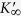 were also fixed to be the values obtained in fit 5. As a result, we obtained the parameters which agree with the present theoretical results, as discuss below. Since 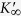 does not satisfy Keane’s rule (*K*_*0*_′/2 < 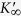 < *K*_*0*_′-1) there is a possibility that *K*_*0*_′ and 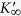 are slightly over- and underestimated, respectively, in fit 7. This fact indicates that the present experimental pressure is still not sufficient to determine 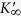 precisely. Nevertheless, there was no significant discrepancy between the obtained parameters in fit 7 and the results from the *ab initio* data (fit 8).

**Figure 2:**
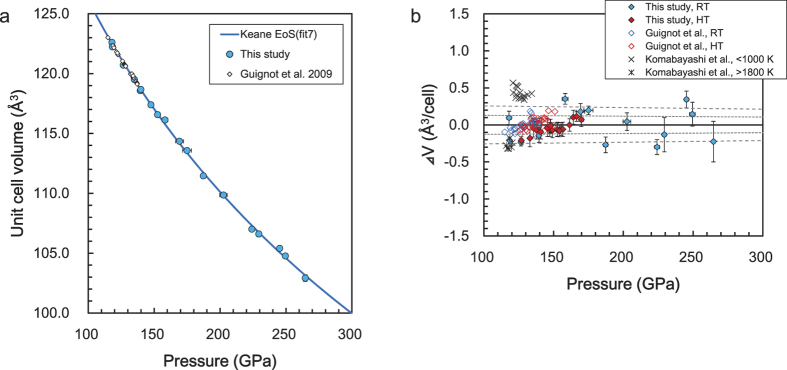
(**a**) The compression curve of MgSiO_3_ post-perovskite. The solid circles represent the present experimental data at 300 K. The open diamonds show the results of Guignot *et al.*^10^. The pressures of all data were calculated based on the Tange-MgO scale in Keane EoS model. The solid curve shows the fitting result using the Keane EoS. (**b**) The residual volume of the data from the EoS. The high temperature data are also plotted. The dotted and dashed lines show a 0.1% and 0.2% volume difference at each pressure condition, respectively.

The compression curve, based on the Keane model (fit 7), is shown in Fig. 2a, along with the experimental data at 300 K. Figure 2b shows the volume difference from the EoS, including both of the data at 300 K and at high temperature. Most of the data are reproduced by the EoS within 0.1% of the volume. The data at over 200 GPa were relatively scattered mainly due to the overlapping of the X-ray diffraction peaks from MgO and gold. If we use the same pressure scale and choose the quasi-hydrostatic data (*St* < 0.005, see Supplementary Information B), there is no discrepancy between the result of this study and that of Guignot *et al.*^10^. The differences between each EoS models are discussed below and in Supplementary Information C.

### Fitting the EoS to the *ab initio* data

In the fitting of the EoS to the present *ab initio* data, RMS(P) for the 3BM, Vinet, AP2, and Keane models were 0.8, 1.0, 0.7, and 0.5 GPa, respectively, for all data, and 1.2, 4.2, 1.9, and 0.4 GPa, respectively, for the data at 300 K. The fitting of the data with the Keane EoS model showed the minimum RMS value. The Keane EoS parameters (fit 8) are shown in Table 1. The results for the other EoS models are summarized in Supplementary Information D, Table S4. Using the theoretical *P-V-T* data, with a wide *P-T* range, we determined all of the parameters to be free parameters, without using either Slater’s formula for *γ* or the equation for *β*. The parameters obtained in fit 8 satisfy both the thermodynamic constraints and Keane’s rule well, and these parameters agree with the experimental results (fit 7).

### The difference between the EoS models, and a comparison with previous studies

**Figure 3:**
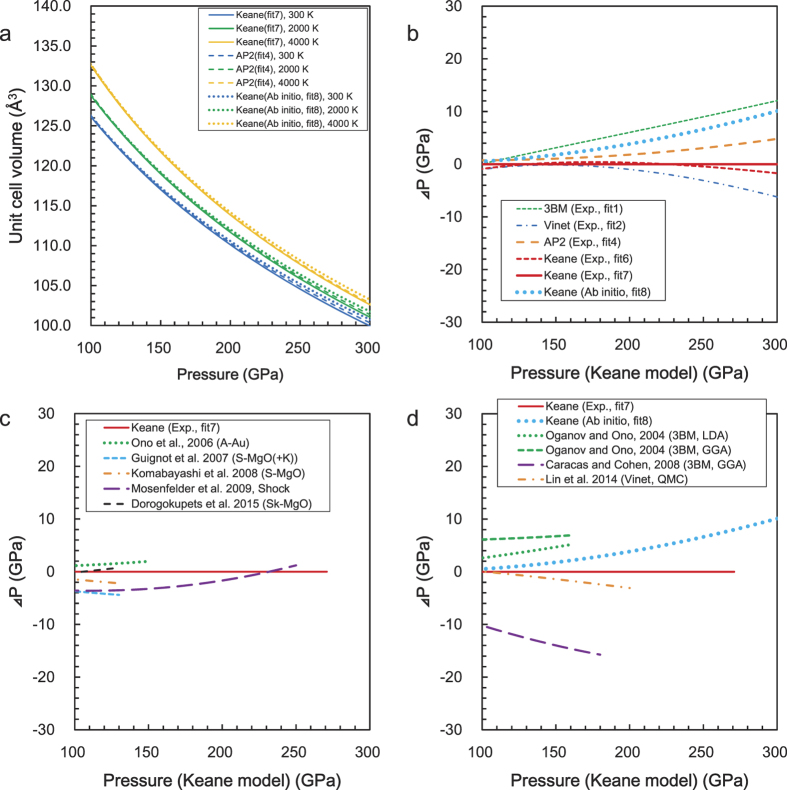
(**a**) The compression curves for the experimental Keane and AP2 EoS and theoretical Keane EoS. (**b**) The pressure differences for the 3BM (fit 1), Vinet (fit 2), AP2 (fit 4) and Keane (fits 6 and 8) with respect to the Keane EoS (fit 7) at 300 K. (**c**) The comparison between fit 7 and the previous experimental works at 300 K. A-Au, Anderson *et al.*’s Au scale^29^; S-MgO, Speziale *et al.*’s MgO scale^21^; K, Karki *et al.*’s MgO scale^30^; Sk-MgO, Sokolova *et al.*’s MgO scale^22^, respectively. (**d**) The comparison between fit 7 and the previous theoretical works at 300 K.

Figure 3a shows the compression curves of the experimental Keane and AP2 EoS, and the theoretical Keane EoS. Here, it should be mentioned that the data, for example around 100 GPa and 4000 K, are fictitious, because the Pv-PPv phase boundary exist at around 119 GPa and 2400 K with a gradient of 11.5 MPa/K^3^. Since the melting temperature of PPv is 6200 K at 142 GPa^20^, the P-T condition shown in Fig. 3a are expected to be in the solid state. The valid and invalid quasi-harmonic approximation (QHA) regions are the same as in Tsuchiya *et al.*^5^.

The pressure differences with respect to fit 7 are shown in Fig. 3b. The difference between fits 6 and 7 was found to be less than 2 GPa in the pressure range from 100 to 300 GPa. This indicates that the difference in *V*_*0*_ does not affect the calculated pressures in this pressure range. The difference in the 3BM, Vinet, and AP2 models were directly affected by the difference in the pressure scale. The *ab initio* calculations yielded a relatively larger pressure, approximately +0.5–3.4%. In the case of high temperatures, the differences were reduced compared to those at 300 K. On the other hand, the volume differences (Δ*V*) at 100–140 GPa and 300–4000 K, with respect to fit 7, fell in only 0.1% of the AP2 EoS (fit 4) and the theoretical Keane EoS (fit 8), and in 0.4% of fit 4 and 0.8% of fit 8, up to 300 GPa and 5000 K. It should be noted that the experimental Keane EoS (fit 7) have their own errors: 1*σ*_*V*_ = 0.1–0.4% and 1*σ*_*P*_ = 1.2–1.9% for the ranges of 100–300 GPa and 300–5000 K, respectively.

Figure 3c shows the comparison between fit 7 and the previous experimental works at 300 K. As shown in Fig. 2, when we use the same pressure scale, the results of this study (fit 7) are fairly consistent with the results obtained by Guignot *et al.*^10^. Therefore, the difference in Fig. 3c shows the difference in the pressure scales; i.e., the difference between Speziale *et al.*’s MgO scale^21^ and the scale-free unified analysis (SFUA)-Keane MgO scale (see Method), at 300 K. Recently, Sokolova *et al.*^22^ proposed the “optimized” pressure scale for MgO. This MgO pressure scale is quite consistent with the present Keane MgO scale at 300 K (see Supplementary Information C). As a result, the latest PPv EoS reported by Dorogokupets *et al.*^23^ is consistent with ours at 100–130 GPa because they used Sokolova *et al.*’s MgO scale. The differences between the present results and those of Komabayashi *et al.*^11^ and Ono *et al.*^9^ may be partially due to the effect of the uniaxial stress. The results obtained by Mosenfelder *et al.*^12^ are consistent with those obtained by Guignot *et al.*^10^ at around 100 GPa, because they used Guignot *et al.*’s *P-V-T* data and *V*_*0*_^10^.

Figure 3d shows the comparison between fit 7 and previous theoretical works at 300 K. The present *ab initio* results (fit 8) show a similar tendency to those of Oganov and Ono^2^. The calculation results of the quantum Monte Carlo (QMC) simulation^7^ are in strong agreement with the present results, at around 100 GPa, although they show a slightly smaller pressure above 200 GPa. On the other hand, the EoS reported by Caracas and Cohen^6^ yields much smaller pressures with respect to the present results.

These acceptable agreements found between the experiment and theory can be regarded as a highly reasonable result. The DFT, with LDA and QHA, is known to work satisfactorily well for the description of the thermal EoS of PPv, while the GGA with QHA predicts an approximately 10 GPa larger thermal pressure than the LDA result, as confirmed in the DFT studies of MgSiO_3_-PPv^2^,^4^,^5^. Lin *et al.*^7^ recently attempted to verify the DFT, with LDA, and GGA formalism for electron many-body effects by applying the QMC technique, which is known as a more stringent treatment for the ground state properties. They then demonstrated that the correction for the static pressure, by the QMC simulation, is quite small for the LDA pressure. As a consequence, our LDA calculation, Oganov and Ono^2^, and the Lin *et al.*^7^’s EoS are, thus, very similar to one another, as confirmed in Fig. 3d. We observed that the present theoretical prediction maintains its excellent agreement with the experiment, with an error of only a few percent, even at a much higher pressure, up to 300 GPa, reaching the conditions of the Earth’s core.

We observed a peculiar discrepancy in the EoS between Caracas and Cohen^6^ and the other prediction (Fig. 3d). The EoS of Caracas and Cohen^6^ was obtained using both the LDA and GGA at the static conditions. Although the GGA result is found to be approximately 10% smaller pressure with respect to the earlier LDA predictions and present result (Fig. 3d), the LDA result (not depicted in Fig. 3d) is found to be a much smaller pressure (about 30% smaller at 100 GPa, and more at higher pressure). The temperature effect may change this situation. However, even if one considered the quantum correction to the free energy and evaluated the thermal pressure, it would be difficult to resolve the discrepancy among theoretical studies because the thermodynamic properties of Mg-PPv at such a low temperature of 300 K are almost the same as those calculated at the static condition^4^.

### The thermal expansion and the Grüneisen parameter

**Figure 4:**
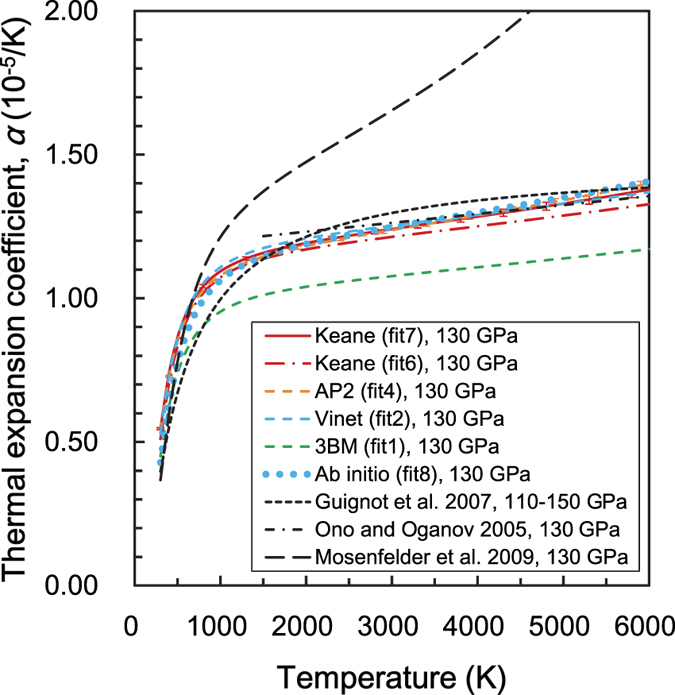
The thermal expansion coefficient at 130 GPa as a function of temperature. The errors associated with the Keane (fit 7) and AP2 models (fit 4) are shown as 1σ, and calculated from each error in the EoS parameters.

The thermal expansion coefficient, at 130 GPa, is shown in Fig. 4. There are no significant differences between fits 6 and 7, despite the fact that their *V*_*0*_s are very different. The theoretical result (fit 8) is fairly consistent with the experimental Keane EoS (fit 7). The Vinet (fit 2) and AP2 (fit 4) EoS agree well, but 3BM (fit 1) shows slightly lower values. The previous theoretical report by Ono and Oganov^8^ is also consistent. Although the difference between fit 7 and the results obtained by Guignot *et al.*^10^ is not significant, their curvature is slightly different due to Guignot *et al.*^10^ use of the original function. On the other hand, the results based on the shock experiment^12^ show much larger values. Conversely, the Keane model, based on LHDAC data, can successfully reproduce the theoretical prediction.

**Figure 5:**
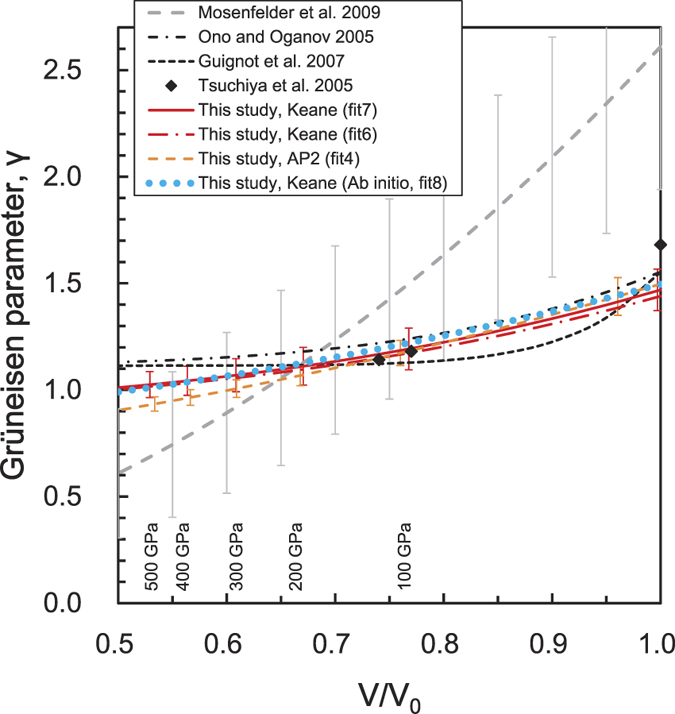
The Grüneisen parameter at 300 K as a function of volume. The errors in the present results and those of Mosenfelder *et al.*^12^ are shown as 1σ.

Figure 5 shows the Grüneisen parameter at 300 K as a function of volume. The present experiment (fit 7) obtained *γ*_*0*_ and *γ*_*∞*_ values of 1.47 and 0.93, respectively. These values are consistent with the theoretical results (fit 8), 1.493 and 0.817 for *γ*_*0*_ and *γ*_*∞*_, respectively. Our experimental and theoretical results show slightly lower values for *γ*_*∞*_ compared to the previous theoretical results^8^. Guignot *et al.*^10^ reported a relatively large value for *β*, using Ono and Oganov’s values^8^ for *γ*_*0*_ and *γ*_*∞*_ (*β* = 13); however, the present experimental results show the value of *β* is reduced by around 2~3. Our experimental values are also lower than the result, *β* = 4.731, reported by Ono and Oganov^8^. On the other hand, the present theoretical calculation was performed over a wide *P-T* range, from −10 GPa and 300 K to 1200 GPa and 5000 K, which yielded a value for *β* of approximately 2, without using the *β – γ*_*0*_*– γ*_*∞*_ relation. Consequently, the volume dependencies of *γ* obtained from the present experimental and theoretical works are quite consistent, as seen in Fig. 5.

## Conclusions

We examined the *P-V-T* relationship of MgSiO_3_ PPv at up to 265 GPa by using a LHDAC experiment, and up to 1200 GPa and 5000 K by the *ab initio* calculation within the DFT. The Keane and AP2 EoS models were newly adapted to this phase in order to establish the thermodynamic EoS at multi-megabar conditions, with the parameters set at infinite pressure. The parameters such as *V*_*0*_, *γ*_*0*_, *γ*_*∞*_, and 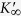 were successfully determined by using data with a wide *P-T* range. The obtained parameters were found to be very consistent between those obtained in the experiment and those obtained by theoretical calculation. Furthermore, these parameters satisfy the rigorous thermodynamical constraints. The present result is the first report of the fully experimentally based Grüneisen parameter using LHDAC data. Its tendency with respect to the volume was fairly consistent with the theoretical prediction.

It has previously been predicted by *ab initio* computations that the PPv phase decomposes above approximately 900–1000 GPa^15^,^24^. The *ab initio* calculation in this study covered the pressure range of 0 to 1200 GPa. The experimental results are applicable up to approximately 300 GPa, which corresponds to the pressure at the core-mantle boundary of a super-Earth with a mass approximately 3 times that of the Earth^24^. The *P-V-T* data of present experimental EoS (fit7) and theoretical EoS (fit8) are summarized in Supplementary Information (Table S5) for the convenience. When the experimental EoS were extrapolated to even higher pressures, the difference between the experimental model and the theoretical model was found to be not significant: Δ*V* < 1.6% up to 500 GPa and 5000 K (and doubled after an extrapolation to 1000 GPa and 5000 K). We strongly believe that the results of this study are considerably useful to discuss the physical properties of not only the Earth’s lowermost mantle, but also a super-Earth’s mantle.

## Method

The choice of the pressure scale is the most important factor for the determination of the EoS. The MgO pressure scale, outlined by Speziale *et al.*^21^, was widely used; Guignot *et al.*^10^ and Komabayashi *et al.*^11^ also used this scale. However, Tange *et al.*^26^ pointed out that this scale cannot reproduce the thermal expansion at 1 atm, and they proposed a primary scale which was based on the scale-free unified analysis (SFUA). Tange *et al.*^26^ reported two sets of parameters for the 3^rd^ order Birch-Murnaghan (3BM) and Vinet EoS models. However, the pressure difference between the 3BM and Vinet EoS reached over 10 GPa above 200 GPa, due to the differences in their EoS formulae. Thus, a more physically applicable EoS model is required for ultra-high pressure conditions. In this study, the Keane^27^,^28^ and AP2^29^ EoS models were adopted as ultra-high pressure EoS models. All the EoS formulae are summarized in Supplementary Information A. The MgO EoS parameters, based on the Keane and AP2 models by the SFUA (Tange, unpublished data), are summarized in Table S3. The key points that should be noted with these EoS are, first, that they include the parameter at infinite pressure (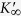 or *a*_*FG*_). Second, these EoS yield an intermediate pressure that lies between 3BM and Vinet (Fig. S3). Third, the number of fitting parameters is same as for the case of 3BM and Vinet, with the MGD model, when the Slater’s formula for the Grüneisen parameter 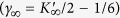 is adopted^16^.

The details of the experimental procedure and the *ab initio* calculation are summarized in Supplementary Information C.

## Additional Information

**How to cite this article**: Sakai, T. *et al.* Experimental and theoretical thermal equations of state of MgSiO_3_ post-perovskite at multi-megabar pressures. *Sci. Rep.* **6**, 22652; doi: 10.1038/srep22652 (2016).

## Supplementary Material

Supplementary Information

Supplementary Table S5

## Figures and Tables

**Table 1:** MgSiO_3_ post-perovskite EoS parmeters.

Formula	Room temperature EoS parmeters			High Temperture EoS parameters								
	V_0_ [Å^3^/cell]	K_0_ [GPa]	K_0_′	γ_0_	γ_∞_	q or β^*a^	θ_0_[K]	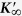 ^*b^	Fit #	memo	P scale	Ref.
Vinet	162.86	231.93	4.430							Ab initio, LDA	—	Oganov and Ono^2^
Vinet	167.64	199.96	4.541							Ab initio, GGA	—	Oganov and Ono^2^
3BM/Vinet	163.81 ± 0.05	222 ± 1	4.2 ± 0.1	1.64	—		1100			Ab initio, LDA	—	Tsuchiya *et al.*^4^
	167.67			1.553	1.114	4.731	—			Ab initio, GGA	—	Ono and Oganov^8^
3BM	162.86	237 ± 1	4.0 fix							Exp., LHDAC	A-Au^*c^	Ono *et al.*^9^
3BM	162.86	248 ± 1	4.0 fix							Exp., LHDAC	Dw-Au^*d^	Ono *et al.*^9^
3BM	162.2	231.2 ± 0.1	4.0	1.553 fix	1.114 fix	13 ± 1	—			Exp., LHDAC	S/K-MgO^*e^	Guignot *et al.*^10^
3BM	163.40	200	4.23							Ab initio, GGA	—	Caracas and Cohen 6
3BM	162.2	225 ± 2	4.21 ± 0.07	2.61 ± 0.67	—	2.1 ± 0.8	990 ± 146			Exp., Shock	—	Mosenfelder *et al.*^12^
3BM	158.0 ± 1.5	292 ± 22	3.74 ± 0.13	1.31 ± 0.10	0.93 fix	3.5 ± 0.5	971 ± 287	—	fit1	Exp., LHDAC	T-MgO^*f^(3BM)	This study +Guignot *et al.*^10^
Vinet	156.5 ± 1.6	324 ± 27	3.30 ± 0.23	1.48 ± 0.09	0.93 fix	2.7 ± 0.2	931 ± 232	—	fit2	Exp., LHDAC	T-MgO^*f^(Vinet)	This study +Guignot *et al.*^10^
AP2	155.9 ± 1.6	336 ± 28	3.13 ± 0.23	1.46 ± 0.09	2/3 fix	1.84 ± 0.08	933 ± 257	—	fit3	Exp., LHDAC	T-MgO(AP2)	This study +Guignot *et al.*^10^
	163.45^*g^ fix	228 ± 1	4.18 ± 0.04	1.50 ± 0.10	2/3 fix	1.80 ± 0.08	849 ± 260	—	fit4	Exp., LHDAC	T-MgO(AP2)	This study +Guignot *et al.*^10^
Keane	157.16 ± 4.54	310 ± 111	3.47 ± 2.35	1.44 ± 0.19	0.93 ± 0.63	2.8 ± 1.6	938 ± 264	2.19 ± 1.27	fit5	Exp., LHDAC	T-MgO(Keane)	This study +Guignot *et al.*^10^
	157.16 fix	310 ± 2	3.47 ± 0.05	1.44 ± 0.09	0.93 fix	2.8 ± 0.3	938 ± 240	2.19 fix	fit6	Exp., LHDAC	T-MgO(Keane)	This study +Guignot *et al.*^10^
	164.26^*g^ **fix**	**203 ± 2**	**5.35 ± 0.09**	**1.47 ± 0.10**	**0.93 fix**	**2.7 ± 0.3**	**848 ± 238**	**2.19 fix**	fit7	Exp., LHDAC	T-MgO(Keane)	This study +Guignot *et al.*^10^
Keane	**164.22 ± 0.06**	**205.4 ± 0.7**	**5.069 ± 0.019**	**1.495 ± 0.005**	**0.818 ± 0.012**	**1.97 ± 0.07**	**995 ± 17**	**2.627 ± 0.002**	fit8	Ab initio, LDA	—	This study

*a: β = γ_0_/(γ_0_ − γ_∞_) except for fit8.

*b: γ_∞_ = 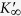/2 − 1/6 or 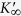 = 2(γ_∞_ + 1/6).

*c: A-Au: Equation of state of Au by Anderson *et al.*^29^ was used as a pressure scale.

*d: Dw-Au: Equation of state of Au by Dewaele *et al.* (2004) was used as a pressure scale.

*e: S/K-MgO: Equation of state of MgO by Speziale *et al.*^21^ was used as a pressure scale for the room temperature data. Thermal pressure was calculated based on Karki *et al.*^30^.

*f: T-MgO: Equation of state of MgO by Tange *et al.*^26^ was used as a pressure scale.

*g: determined by the g-G plot.
