# Supplementary material for: Experimental and theoretical thermal equations of state of MgSiO3 post-perovskite at multi-megabar pressures
Source: Sci Rep. 2016 Mar 7;6:22652. doi: 10.1038/srep22652 (PMC4780068; doi:10.1038/srep22652)
Supplement: Supplementary Information [file srep22652-s1.pdf]

Supplementary Information

Experimental and theoretical thermal equations of state of $MgSiO_3$ post-perovskite at multi-megabar pressures

Takeshi Sakai[1*], Haruhiko Dekura[1], Naohisa Hirao[2]

1. Geodynamics Research Center, Ehime University, Matsuyama 790-8577, Japan

2. Japan Synchrotron Radiation Research Institute, Hyogo 679-5198, Japan

*: corresponding author

E-mail: sakai.takeshi.mk@ehime-u.ac.jp

This file includes:

- Supplementary Information A: All the EoS formulae
- Supplementary Information B: Result of XRD and the axial compressibility

  Figure S1, Figure S2, Table S1, Table S2
- Supplementary Information C: Method

  Figure S3, Table S3
- Supplementary Information D: Results of EoS fitting for the *ab initio* data

  Table S4
- References in Supplementary Information

Supplementary Information A: All the EoS formulae

Third order Birch-Murnaghan (3BM) EoS [31]

$$\mathrm{P}=\frac{3}{2}K_0\left[\left(\frac{V}{V_0}\right)^{-\frac{7}{3}}-\left(\frac{V}{V_0}\right)^{-\frac{5}{3}}\right]\left\{1+\frac{3}{4}(K_0'-4)\left[\left(\frac{V}{V_0}\right)^{-\frac{2}{3}}-1\right]\right\}$$

Vinet (Morse-Rydberg) EoS [32]

$$\mathrm{P}=3K_0\left(\frac{V}{V_0}\right)^{-\frac{2}{3}}\left[1-\left(\frac{V}{V_0}\right)^{\frac{1}{3}}\right]exp\left\{\frac{3}{2}(K_0'-1)\left[1-\left(\frac{V}{V_0}\right)^{\frac{1}{3}}\right]\right\}$$

where V_0, K_0, and K_0' are the zero pressure volume, bulk modulus, and the bulk modulus pressure derivative, respectively.

Adapted Polynomial expansion of 2nd order (AP2) EoS [28]

$$P=3K_0\frac{1-x}{x^5}exp\{C_0(1-x)\}\{1+xC_2(1-x)\}$$

$$x=\left(\frac{V}{V_0}\right)^{\frac{1}{3}}$$

$$C_0=-\ln\frac{3K_0}{p_{FG_0}}$$

$$p_{FG_0}=a_{FG}\left(\frac{Z_e}{V_0}\right)^{\frac{5}{3}}$$

$$a_{FG}=2336.965\ [GPa\cdot\text{Å}^5]$$

$$C_2=\frac{3}{2}(K_0'-3)-C_0$$

p_{FG_0} and a_{FG} are the Fermi gas pressure and the universal Fermi gas parameter, respectively. Z_e is the total number of electrons in the unit cell with a V_0 ($Z_e = (12 + 8) \times 4 = 80$ for MgO, and $Z_e = (12 + 14 + 8 \times 3) \times 4 = 200$ for $MgSiO_3$ in the case of the V_0 units being [Å^3/cell]). Stacey and Davis[16] reported that AP2 EoS yields $K'_\infty = \frac{5}{3}$, where K'_∞ is the pressure derivative of the bulk modulus at infinite pressure. The K'_∞ should be $K'_\infty > \frac{5}{3}$ but Stacey[33] argued that "there is a fundamental significance to the value $\frac{5}{3}$ as the minimum possible value of K'_∞ for solids". This minimum K'_∞ yields $\gamma_\infty = \frac{2}{3}$ from Slater's formula (see below). Thus, γ_∞ was fixed to be $\frac{2}{3}$ for the AP2 EoS in this study.

Keane EoS [16,27]

$$\frac{P}{K_0} = \frac{K'_0}{{K'_\infty}^2}\left[\left(\frac{V_0}{V}\right)^{K'_\infty} - 1\right] - \left(\frac{K'_0}{K'_\infty} - 1\right)\ln\left(\frac{V_0}{V}\right)$$

Keane's rule is expressed: $K'_0 - 1 > K'_\infty > K'_0/2$.

Mie-Grüneisen-Debye (MGD) model

$$P\,(V,T) = P(V,\,T)_{T=300K} + \Delta P_{th}(V,T)$$

$$\Delta P_{th}(V,T) = E_{th}(V,T) - E_{th}(V,\,T)_{T=300K}$$

$$E_{th}(V,T) \approx 9nRT\frac{\gamma}{V}\left(\frac{T}{\theta_D}\right)^3 \int_0^{\frac{\theta_D}{T}} \frac{z^3}{e^z - 1} dz$$

where n is the number of atoms in the formula unit of the concerned material ($n = 2$ for MgO, and $n = 5$ for $MgSiO_3$ post-perovskite), R is the gas constant, γ is the Grüneisen parameter, and θ_D is the Debye temperature.

Gruneisen parameter and Debye temperature in Al'tshuler et al.'s form [13]

$$\gamma = \gamma_\infty + (\gamma_0 - \gamma_\infty)\left(\frac{V}{V_0}\right)^\beta$$

$$\theta_D = \theta_0\left(\frac{V}{V_0}\right)^{-\gamma_\infty} exp\left\{\frac{\gamma_0 - \gamma_\infty}{\beta}\left[1 - \left(\frac{V}{V_0}\right)^\beta\right]\right\}$$

$$\beta = \frac{\gamma_0}{\gamma_0 - \gamma_\infty}$$

Here, the subscript 0 and ∞ mean the ambient and infinite pressure and temperature conditions, respectively. Note that the parameter β is sometimes used as a free parameter[19,26,34]. In this study, the relation between β, γ_0, and γ_∞ was only used for the EoS fitting to the experimental data of $MgSiO_3$ post-perovskite.

Slater's formula for γ [16,35]

$$\gamma_\infty = \frac{K'_\infty}{2} - \frac{1}{6}$$

This formula is obtained by expressing the original formula ($\gamma_s = K'/2 - 1/6$) at infinite pressure. The thermodynamical constrains for K'_∞ and γ_∞ were obtained using

a combination of this relation and the relation between K'_∞ and γ_∞ ($K'_\infty > 1 + \gamma_\infty$), as follows[16]:

$$K'_\infty > \frac{5}{3}, \qquad \gamma_\infty > \frac{2}{3}$$

Supplementary Information B: Result of XRD and the axial compressibility

X-Ray diffraction patterns

Representative X-ray diffraction patterns are shown in Figure S1. The diffraction peaks of PPv, MgO, and Au were observed up to pressures of 170 GPa at a temperature of 2560 K, and 256 GPa at 300 K. All data at room temperature were obtained after the laser annealing over 1500 K. Using the observed diffraction peaks of MgO, we calculated the St value in order to evaluate the effect of the uniaxial stress. Here, S is the elastic anisotropy factor, and t is the uniaxial stress. The effect of the uniaxial stress on the volume is thought to be small when a quasi-hydrostatic condition, $St < 0.005$, is satisfied. The St values can be calculated from the so-called "Γ plot"[36]. We used only the data with $St < 0.005$ for the EoS fitting. The data obtained by Guignot et al.[10] were also included for the EoS fitting. The unit cell parameters are summarized in Table S1.

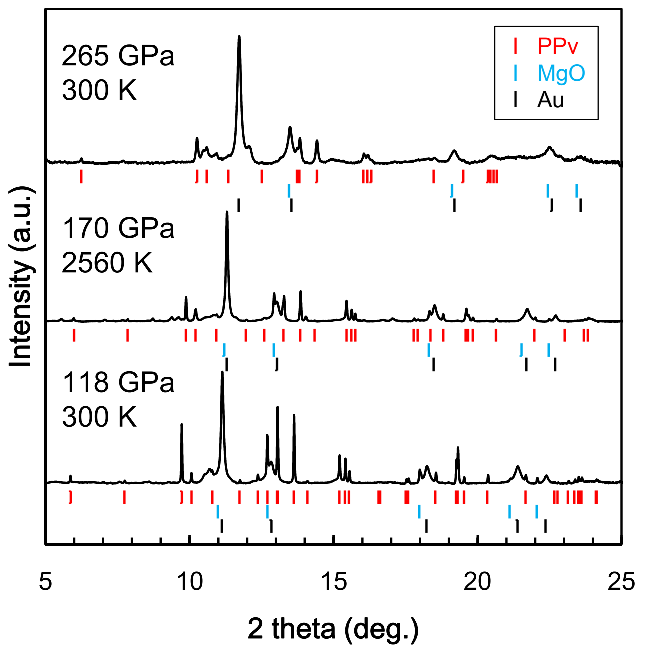

Figure S1. Representative X-ray diffraction patterns of $MgSiO_3$ post-perovskite (PPv). Red, blue, and black bars show the observed diffraction peak positions for PPv, periclase (MgO), and gold (Au), respectively.

Table S1. Experimental P-T conditions and cell parameters.

Run	Pressure				Temperature	$MgSiO_3$ cell parameters				MgO cell parameters	
	Keane	AP2	3BM	Vinet							
	(GPa)	(GPa)	(GPa)	(GPa)	(K)	a (Å)	b (Å)	c (Å)	V (Å^3)	a (Å)	V (Å^3)
PP02_004	118.0(15)	118.7(15)	119.5(16)	117.9(15)	300	2.4691(5)	8.0997(16)	6.1306(11)	122.61(9)	3.745(2)	52.54(14)
PP02_006	118.4(4)	119.1(4)	119.9(5)	118.3(5)	300	2.4679(3)	8.0898(9)	6.1224(5)	122.23(4)	3.745(1)	52.51(3)
PP02_011	126.6(3)	127.4(3)	128.4(5)	126.4(5)	300	2.4573(3)	8.0516(7)	6.1004(7)	120.70(5)	3.7273(4)	51.78(3)
PP02_016	135.0(17)	136.0(17)	137.2(19)	134.7(18)	300	2.4498(3)	8.0178(11)	6.0838(9)	119.50(6)	3.710(2)	51.08(14)
PP02_023	139.5(6)	140.5(6)	141.9(8)	139.2(7)	300	2.4451(5)	7.9901(15)	6.0693(14)	118.57(9)	3.702(1)	50.72(4)
PP02_021	139.9(12)	140.9(13)	142.3(14)	139.6(13)	300	2.4448(4)	7.9942(10)	6.0723(9)	118.68(6)	3.701(2)	50.69(10)
PP02_028	147.6(6)	148.7(6)	150.3(8)	147.2(8)	300	2.4367(6)	7.9586(16)	6.0534(13)	117.39(9)	3.686(1)	50.10(5)
PP02_029	152.6(7)	153.9(7)	155.6(9)	152.2(9)	300	2.4307(6)	7.9401(16)	6.0394(11)	116.56(9)	3.677(1)	49.72(5)
PP02_033	158.2(21)	159.6(22)	161.5(23)	157.7(22)	300	2.4280(4)	7.9268(13)	6.0336(10)	116.12(7)	3.667(2)	49.32(15)
PP01_008b	169.3(30)	170.8(31)	173.2(33)	168.6(30)	300	2.4180(6)	7.8691(23)	6.0095(12)	114.34(11)	3.649(3)	48.57(20)
PP01_009	174.9(33)	176.6(33)	179.2(35)	174.1(33)	300	2.4120(4)	7.8513(10)	5.9973(8)	113.57(6)	3.639(3)	48.20(21)
PP01_013	187.3(16)	189.1(16)	192.3(18)	186.3(17)	300	2.3964(6)	7.7952(19)	5.9663(15)	111.45(11)	3.620(1)	47.43(9)
PP01_017	202.6(28)	204.7(28)	208.5(31)	201.2(28)	300	2.3859(7)	7.7532(22)	5.9384(17)	109.85(12)	3.597(2)	46.55(16)
PP01_026b	224.2(18)	226.6(18)	231.6(21)	222.3(19)	300	2.3654(6)	7.6813(19)	5.8894(14)	107.01(10)	3.568(1)	45.41(9)
PP01_029	229.4(13)	232.0(14)	237.2(17)	227.4(16)	300	2.3632(11)	7.6643(46)	5.8853(31)	106.60(23)	3.561(1)	45.14(7)
PP01_034	245.3(15)	248.1(15)	254.4(19)	242.8(17)	300	2.3570(6)	7.6315(21)	5.8595(17)	105.40(11)	3.541(1)	44.39(7)
PP01_037	249.5(20)	252.5(20)	259.0(24)	246.9(22)	300	2.3528(11)	7.6041(26)	5.8559(25)	104.77(16)	3.535(1)	44.19(9)
PP01_041	264.6(12)	267.8(12)	275.3(18)	261.5(16)	300	2.3386(16)	7.5629(53)	5.8191(38)	102.92(27)	3.518(1)	43.53(5)
PP02_010	135.5(9)	136.2(9)	136.5(17)	135.6(17)	1,670(30)	2.4604(7)	8.0530(20)	6.1070(18)	121.00(12)	3.729(1)	51.83(6)
PP02_032	164.1(8)	165.3(8)	166.4(18)	163.9(18)	1,680(30)	2.4302(5)	7.9339(17)	6.0393(13)	116.45(9)	3.674(1)	49.60(4)
PP02_020	147.2(6)	148.0(6)	148.6(16)	147.1(18)	1,760(30)	2.4489(5)	8.0015(16)	6.0775(14)	119.09(9)	3.7067(4)	50.93(3)

Table S1 (*continued*)

Run	Pressure				Temperature	$MgSiO_3$ cell parameters				MgO cell parameters	
	Keane	AP2	3BM	Vinet							
	(GPa)	(GPa)	(GPa)	(GPa)	(K)	a (Å)	b (Å)	c (Å)	V ($Å^3$)	a (Å)	V ($Å^3$)
PP02_027	155.4(7)	156.4(7)	157.1(17)	155.3(19)	1,780(30)	2.4392(5)	7.9687(14)	6.0594(13)	117.78(9)	3.692(0)	50.31(3)
PP02_009	137.7(11)	138.3(11)	138.4(20)	137.8(20)	2,050(70)	2.4613(7)	8.0548(20)	6.1092(18)	121.12(12)	3.730(1)	51.90(5)
PP02_026	156.6(8)	157.6(9)	158.1(20)	156.6(22)	2,080(50)	2.4404(6)	7.9735(17)	6.0614(13)	117.95(10)	3.694(1)	50.39(3)
PP02_019	149.2(6)	150.0(6)	150.3(19)	149.2(21)	2,090(40)	2.4498(6)	8.0030(17)	6.0787(15)	119.18(10)	3.7079(3)	50.98(2)
PP02_031	166.5(10)	167.6(10)	168.3(22)	166.4(23)	2,110(50)	2.4326(4)	7.9330(12)	6.0431(7)	116.62(6)	3.676(1)	49.68(4)
PP02_014	145.5(5)	146.2(5)	146.4(19)	145.6(22)	2,180(50)	2.4538(7)	8.0250(21)	6.0918(19)	119.96(12)	3.7164(1)	51.33(1)
PP02_003	126.9(11)	127.3(11)	127.0(21)	127.1(22)	2,280(40)	2.4760(3)	8.1124(10)	6.1400(7)	123.33(5)	3.757(1)	53.03(7)
PP02_013	147.7(6)	148.4(6)	148.3(22)	147.9(26)	2,530(70)	2.4543(5)	8.0282(15)	6.0942(13)	120.07(9)	3.71751(4)	51.376(2)
PP02_030	169.9(15)	170.9(15)	171.4(28)	169.9(28)	2,560(90)	2.4325(6)	7.9302(18)	6.0448(11)	116.60(9)	3.677(1)	49.70(5)
PP02_018	152.7(8)	153.5(8)	153.4(24)	152.9(27)	2,600(70)	2.4503(5)	8.0059(16)	6.0800(14)	119.27(10)	3.7088(3)	51.02(2)
PP02_025	161.4(6)	162.3(6)	162.5(24)	161.5(28)	2,610(40)	2.4407(5)	7.9718(15)	6.0602(11)	117.91(8)	3.6925(4)	50.35(2)
PP02_005	133.0(8)	133.4(8)	133.0(23)	133.3(26)	2,670(60)	2.4729(6)	8.0939(19)	6.1336(17)	122.77(11)	3.7502(5)	52.74(3)
PP02_022	154.1(10)	154.9(10)	154.9(25)	154.3(28)	2,670(100)	2.4491(4)	8.0004(13)	6.0785(12)	119.10(8)	3.7071(2)	50.95(1)
PP02_008	140.8(14)	141.3(14)	141.0(26)	141.0(27)	2,710(90)	2.4640(5)	8.0630(15)	6.1141(14)	121.47(9)	3.7344(9)	52.08(6)

The numbers in parentheses are uncertainties in the last digit.

Axial compressibility

Figure S2a shows the axial compressibility at 300 K plotted with the results of the *ab initio* calculation at static (0 K) conditions. The lattice parameters *a*, *b*, and *c* were fitted using a modified Birch-Murnaghan (mod. BM) EoS[37], expressed as $P = \frac{3}{2} K_{l0} \left[\left(\frac{l}{l_0}\right)^{-7} - \left(\frac{l}{l_0}\right)^{-5} \right] \{1 + \frac{3}{4} (K'_{l0} - 4)\}[\left(\frac{l}{l_0}\right)^{-5} - 1]$, where l_0, K_{l0}, and K'_{l0} are the zero pressure length, incompressibility, and the pressure derivative of the incompressibility for the *l* axis (*l* = *a*, *b*, *c*). The subscript zero denotes the ambient conditions, which are set to 0 GPa and 300 K. For high temperature data, the high temperature lattice parameter $l_T = l_0 exp\left(\int_0^T \alpha(t)\, dt\right), \mathrm{\alpha(t) = a + bt}$ and the high temperature incompressibility $K_T = K_{l0} + \frac{dK}{dT}(T - 300)$ were adopted. Here, *dK/dT* was fixed to be -0.015 $\mathrm{GPa.K^{-1}}$ (Guignot et al.[10]). The obtained parameters are summarized in Table S2.

The *b* axis is found to be the most compressible: 1.9 and 2.4 times more compressible than the *a* and *c* axes, respectively, at 0 GPa and 300 K, and 1.3 and 1.4 times more compressible than the *a* and *c* axes, respectively, at 136 GPa and both 300 K and 3000 K, respectively. The compressibility differences among the axes decrease with increasing pressure, and are not affected by the temperature. As shown in Figure 2a, the present experimental result for each axis approximately agrees with the present result of the *ab initio* calculations. The present experimental results also show a slightly smaller K_{l0} value for the *c* axis than those reported by the previous study, and by the calculation in this study. The experimental incompressibility of $MgSiO_3$ for each axis agrees with that of $Mg_{0.85}Fe_{0.15}Al_{0.15}Si_{0.85}O_3$ PPv[37], within the uncertainty. This indicates that the effect of the iron and aluminum component on the axial compressibility is small.

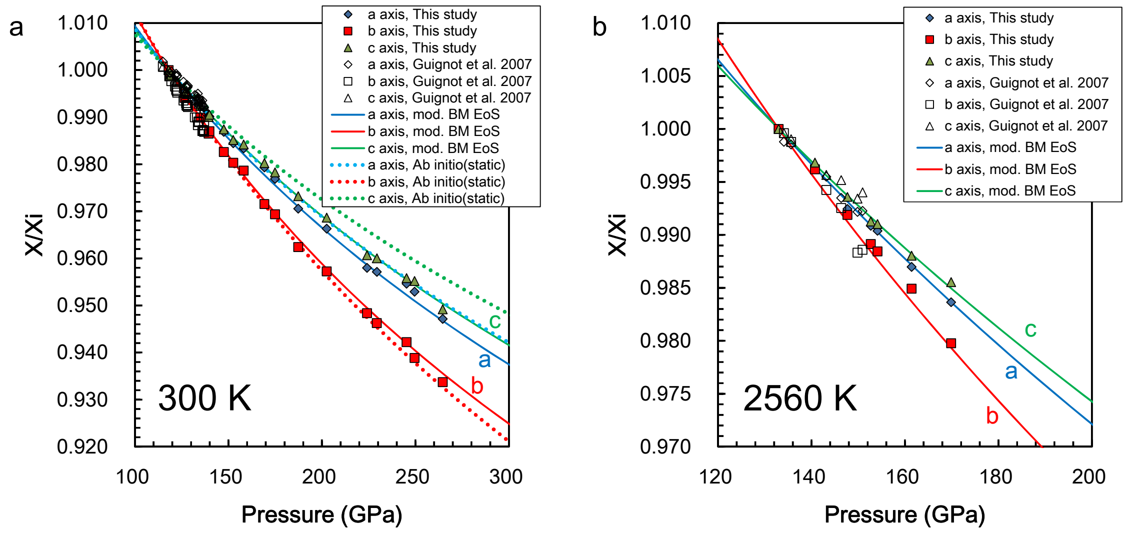

Figure S2. Axial compressibilities at (a) 300 K and (b) 2560 K. Curves colored in blue, red, and green show the fitting results of the modified BM equation for the *a*, *b,* and *c* axes, respectively. The solid and dotted curves represent the experimental and theoretical results, respectively. All of the data are plotted as a relative change with pressures of (a) 118 GPa and (b) 133 GPa. The temperatures in (b) are in the range of 2530-2670 K for the present data and 2438-2520 K for the data taken from Guignot et al.[10]

Table S2. The parameters of a modified Birch-Murnaghan EoS for post-perovskite phase

Composition	Axis	l_0	K_{l0}	K_{l0}'	a [10^{-5} K^{-1}]	b [10^{-8} K^{-2}]	dK/dT	ref.
$MgSiO_3$	***a***	2.703(4)	256(6)	4.0(fix)	0.58(5)	0.10(4)	-0.015(fix)	This study + Guignot et al. 2007
	b	9.248(16)	132(3)	4.0(fix)	1.10(5)	0.25(4)	-0.015(fix)	This study + Guignot et al. 2007
	c	6.630(7)	313(5)	4.0(fix)	0.54(4)	0.05(3)	-0.015(fix)	This study + Guignot et al. 2007
	a	2.68(2)	285(26)[a]	4.0(fix)	-[b]	-[b]	-0.015(fix)	Guignot et al. 2007
	b	9.35(6)	110(9)[a]	4.0(fix)	-[b]	-[b]	-0.015(fix)	Guignot et al. 2007
	c	6.53(2)	403(24)[a]	4.0(fix)	-[b]	-[b]	-0.015(fix)	Guignot et al. 2007
	a	2.687(3)	253(5)	4.47(2)	-	-	-	This study, Ab initio (static, 0 K)
	b	9.148(17)	160(3)	3.64(1)	-	-	-	This study, Ab initio (static, 0 K)
	c	6.554(9)	347(9)	4.53(3)	-	-	-	This study, Ab initio (static, 0 K)
$(Mg_{0.9},Fe_{0.1})SiO_3$	***a***	2.701(11)	254(17)	4.0(fix)	-	-	-	Nishio-Hamane and Yagi, 2009
	b	9.145(53)	153(12)	4.0(fix)	-	-	-	Nishio-Hamane and Yagi, 2009
	c	6.686(14)	273(14)	4.0(fix)	-	-	-	Nishio-Hamane and Yagi, 2009
$Mg_{0.85}Fe_{0.15}Al_{0.15}Si_{0.85}O_3$	***a***	2.703(8)	275(15)	4.0(fix)	-	-	-	Nishio-Hamane and Yagi, 2009
	b	9.318(66)	122(11)	4.0(fix)	-	-	-	Nishio-Hamane and Yagi, 2009
	c	6.674(19)	297(15)	4.0(fix)	-	-	-	Nishio-Hamane and Yagi, 2009

a : Calculated from the linear incompressibility (β); $\beta=3K_{l0}$

b : They used the different formula; $\alpha=\alpha_\infty exp(-b/(1+T))$.

The numbers in parentheses are uncertainties in the last digit.

Supplementary Information C: Method

Experimental procedure

Mg_2SiO_4 forsterite was used as a starting material[38]. High pressure was generated by using a symmetrical diamond anvil cell. Culet sizes of the diamond anvils of 35 and 100 μm were used. The sample was coated by gold, which is a laser absorber, and loaded into a sample hole that had been drilled in a precompressed rhenium gasket. Enstatite glass was used as both a thermal insulator and a pressure medium. Mg_2SiO_4 forsterite decomposes to $MgSiO_3$ PPv and MgO periclase at high pressure, high temperature conditions. Here, MgO was used as a pressure scale material (Table S3). The pressure differences between each EoS models in Table S3 are shown in Figure S3. The AP2 and Keane EoSs yield an intermediate pressure that lies between the 3BM and Vinet. The MgO pressure scale reported by Speziale et al.[21] shows a relatively lower pressure. The "optimized" pressure scale proposed by Sokolova et al.[22] is quite consistent with the Keane EoS. The pressure difference is within 0.4 GPa up to 300 GPa at 300K, although it reaches about 5 GPa at 261 GPa and 4000 K. The difference at high temperature condition is only 2% but it might be caused by the difference of the treatment for the thermodynamic function (Debye or Einstein model) and/or for the function for the Grüneisen parameter in Sokolova et al.[22]

The unit cell volume of the sample was determined by the synchrotron X-ray diffraction experiment at the SPring-8 BL10XU beamline, in Japan. A monochromatic incident X-ray beam, with wavelengths of 0.41502–0.41530 Å, was focused to a diameter of less than 10 μm[39]. The details of the measurement, the calibration method, and the laser heating method are all same as those detailed by Sakai et al.[19]

The pressure errors are calculated from the measured MgO volume error and the temperature error. The temporal fluctuation of the temperature is summarized in Table 1. The spatial fluctuations in the area of 10 μm at the center (corresponding to the X-ray size) were within approximately 150 K.

Table S3. The parameters of MgO equation of state.

	unit	3BM	Vinet	AP2	Keane
V_0	(Å^3)	74.698	74.698	74.698	74.698
K_0	(GPa)	160.64	160.63	160.634	160.622
K_0'		4.221	4.367	4.312	4.3992
n		2	2	2	2
γ_0		1.431	1.442	1.4375	1.445
γ_∞		1.016	1.243	1.175	1.180
β		3.5	5.4	4.6	4.1
θ_0		761	761	761	761
Z		4	4	4	4
Z_e(total electron)		-	-	20	-
a_{FG}	(GPa Å^5)	-	-	2336.91	-
p_{FG_0}	(GPa)	-	-	2619.85	-
K_∞'		-	-	-	2.69

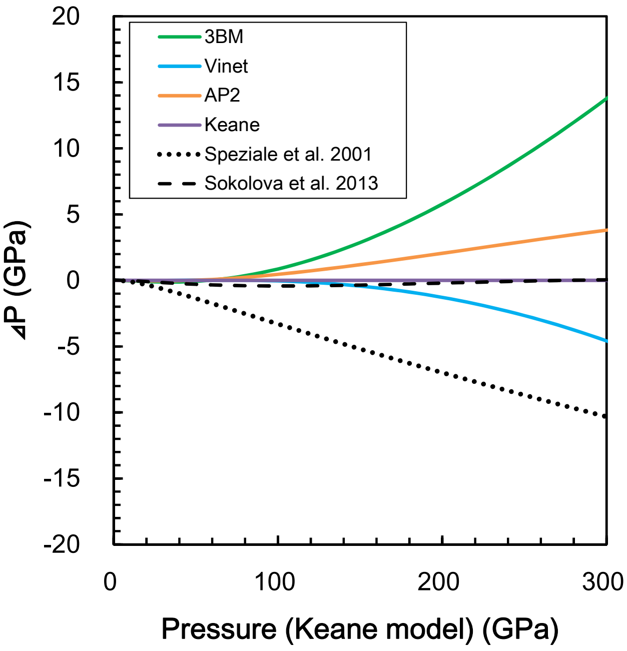

Figure S3. Pressure difference between each EoS models for MgO with respect to the Keane EoS[27] at 300 K. Green and blue curves are the 3BM and Vinet[26], Orange and purple curves are the AP2 and Keane models (Tange, unpublished), respectively. Dotted curve is Speziale et al.[21] Dashed curve is Sokolova et al.[22]

Ab initio calculation

Our *ab initio* calculations were performed based on the density-functional theory (DFT)[40,41], within the local density approximation (LDA)[42,43]. The details of the theoretical method for the total energy calculations are essentially the same as those outlined in previous studies[4,5,44]. Electronic wave functions were expanded by the plane-wave basis set, using norm-conserving pseudopotentials: the von Barth-Car type[30] for magnesium atoms, and Troullier-Martins type[45] for oxygen and silicon atoms. These pseudopotentials were well tested in several studies[4,5,30,44]. The DFT-LDA electronic band calculations were carried out using the QUANTUM ESPRESSO distribution[46]. A plane-wave cut-off of 70 Rydberg was used. We applied the Monkhorst-Pack scheme[47] to sample on 4 × 4 × 2 $\boldsymbol{k}$-point grids in the first Brillouin zone. The structural optimization was performed until the residual force and stress became less than 0.1 $\frac{\text{meV}}{\text{Å.atom}}$ and 0.01 GPa, respectively. We found that these computational conditions yield sufficient convergence for total energies of less than 0.04 $\frac{\text{meV}}{\text{atom}}$ in a more stringent condition with a plane-wave cut-off of 400 Rydberg and with a 12 × 12 × 6 $\boldsymbol{k}$-point grid.

Phonon frequencies were calculated based on the density-functional perturbation theory (DFPT)[48]. In order to generate the dynamical matrices, 4 × 4 × 2 $\boldsymbol{q}$-point grids were sampled. The matrices on the denser $\boldsymbol{q}$-point grids of a 20 × 20 × 20 mesh were computed by using the Fourier-interpolation scheme, in order to obtain convergence in the phonon density of states (PHDOS), $D(\omega)$, defined using the Dirac δ-function as $D(\omega) = \sum_\lambda \delta(\omega - \omega_\lambda)/N_{mode}$, where ω_λ and N_{mode} are the phonon angular frequency of a phonon mode labeled by λ, and the total number of phonon modes of the system, respectively. The integral over the first Brillouin zone was performed using the

linear tetrahedron method for a preferred convergence[49]. The obtained PHDOS were then applied to the calculations of the Helmholtz lattice free energies (F) of the crystals. This will be discussed later in this report.

A static P-V relation was obtained for volumes sampled at pressures of -10, -7, -3, -0.07, 0, 10, 20, 30, 40, 50, 60, 70, 80, 90, 100, 120, 150, 180, 210, 250, 280, 320, 360, 400, 450, 500, 550, 600, 660, 700, 780, 840, 960, 1050, and 1200 GPa. The temperature effect on the pressure was obtained by the derivatives of F with respect to a volume (V) at a constant temperature (T): $P(V,T) = -\left(\frac{\partial F(V,T)}{\partial V}\right)_T$. The free energies, $F(V,T)$, were calculated based on the quasi-harmonic approximation (QHA), which is well known to give accurate descriptions of thermodynamic properties of many materials at high pressures and temperatures[50]. According to the QHA, the free energy, F^{QHA}, is given by $F^{\mathrm{QHA}}(V,T) = E_{static}(V) + F_{electron}(V,T) + F_{phonon}^{\mathrm{QHA}}(V,T)$, where $E_{\mathrm{static}}(V)$ is the adiabatic potential of a static lattice (i.e., the internal energy), $F_{electron}(V,T)$ is the thermal electronic energy, arising approximately from the Kohn-Sham band electronic excitations, neglected in our insulating system, and $F_{phonon}^{\mathrm{QHA}}(V,T)$ is the free energy of phonon within the QHA, which is expressed as

$$F_{phonon}^{QHA}(V,T) = \frac{1}{2}\sum_{\lambda} \hbar\omega_{\lambda}(V) + k_B T \sum_{\lambda} \ln\{1 - \exp(-\frac{\hbar\omega_{\lambda}(V)}{k_B T})\}, \quad (1)$$

where k_{B} and $\hbar$ are, respectively, Boltzmann and Dirac constants. The thermal pressure within the QHA, P_{th}^{QHA}, is then obtained by

$$P_{th}^{\mathrm{QHA}}(V,T) = -\left(\frac{\partial\{F^{\mathrm{QHA}}(V,T) - E_{static}(V)\}}{\partial V}\right)_T = -\left(\frac{\partial\{F_{electron}(V,T) + F_{phonon}^{\mathrm{QHA}}(V,T)\}}{\partial V}\right)_T \approx -\left(\frac{\partial F_{phonon}^{\mathrm{QHA}}(V,T)}{\partial V}\right)_T. \quad (2)$$

In this treatment, only the phonon free energy contributes to the temperature effect on the pressure.

We should point out the fundamental limitation to the use of the QHA for description of the lattice anharmonicity of solids under extremely high *T* conditions. Breaking down of the QHA at very high *T* basically affects the lattice free energy and thus the *ab initio* EoS, most likely making fails in the prediction. Therefore, the realistic EoS might be modified by adding anharmonic corrections to the free energy by taking into account intrinsic phonon-phonon interactions by several things such as molecular dynamics[51], perturbative treatment for the phonon self-energy[52], thermodynamic integration under the virtual Hamilton dynamics[53], and so forth. Those treatments, however, are known to be very computationally demanding. In order to keep satisfactory discussions on the EoS at high *T* in this study, we limited it up to 5000 K in all of the discussions.

We calculated the volume derivatives of F from 300 to 5000 K by a 100 K step by the following numerical process. The derivatives on the unequally spaced grids of volumes, V_i, can be approximated to a divided difference, by the use of the three-point method in Newton's interpolation formula[54]:

$$P(V_i,T) \equiv -\left(\frac{\partial F(V,T)}{\partial V}\right)_{V=V_i} \approx \frac{r_i}{1+r_i}\left(\frac{F(V_{i+1},T)-F(V_i,T)}{V_{i+1}-V_i}\right) + \frac{1}{r_i(1+r_i)}\left(\frac{F(V_i,T)-F(V_{i-1},T)}{V_{i+1}-V_i}\right),$$

(3)

where r_i is defined by $r_i \equiv \frac{V_i-V_{i+1}}{V_{i+1}-V_i}$. This scheme of numerical derivatives ensures an approximate quadratic convergence. We tested also the five-point formula to check the numerical accuracy of the derivatives:

$$P(V_i,T) \approx \frac{\alpha_i\, F(V_{i+2},T) + F(V_{i+1},T) + \beta_i F(V_i,T) + \gamma_i\, F(V_{i-1},T) + \xi_i\, F(V_{i-2},T)}{\delta'_i}$$

$\delta'_i = (\frac{(1+b_i)(1+a_i+b_i)c_i}{b_i\,(a_i+b_i)\,(1+c_i)})\delta_i$, $\delta_i = V_{i+1} - V_i$, $a_i = \frac{V_{i-1}-V_{i-2}}{\delta_i}$, $b_i = \frac{V_i - V_{i-1}}{\delta_i}$, $c_i = \frac{V_{i+2}-V_{i+1}}{\delta_i}$,

$\alpha_i = -\frac{(1+b_i)\,(1+a_i+b_i)}{(1+c_i)^2\,(1+b_i+c_i)\,(1+a_i+b_i+c_i)}$, $\gamma_i = -\frac{(1+a_i+b_i)c_i}{a_i\,b_i^2\,(1+b_i+c_i)}$, $\xi_i = \frac{(1+b_i)c_i}{a_i\,(a_i+b_i)^2\,(1+a_i+b_i+c_i)}$,

$$\beta_i = -\frac{(1+b_i)\,(1+a_i+b_i)\,c_i\,(a_i\,(-1-c_i+b_i\,(2+c_i))+b_i\,(-2\,(1+c_i)+b_i\,(2+c_i)))}{{b_i}^2\,(a_i+b_i)^2\,(1+c_i)^2} \quad .$$

(4)

We found a very small variation in the pressures relative to those calculated by the three-point formula; the maximum error was found to be less than 1**%**. Therefore, we adopted the three-point method (Eq. (3)) for all of the calculations in this study.

Note that these formulas are reduced to well-known simple forms when the volume grid is uniformly spaced, $\delta_i = V_{i+1} - V_i = const. = \delta$; therefore

$P(V_i,T) \approx \left(\frac{F(V_i+\delta,T)-F(V_i-\delta,T)}{2\delta}\right) + O(\delta^2)$ and

$P(V_i,T) \approx \left(\frac{-F(V_i+2\delta,T)+8F(V_i+\delta,T)-8F(V_i-\delta,T)+F(V_i-2\delta,T)}{12\delta}\right) + O(\delta^4)$ for the three- and five-point methods, respectively.

Supplementary Information D: Results of EoS fitting for the *ab initio* data

The EoS parameters for the *ab initio* data are summarized in Table S4. The 3BM yields relatively smaller V_0, higher K_0, and smaller K_0', respectively. As a result, the fitting parameters for 3BM model agree with the previous experimental result[9,10]. In this case, the difference between the 3BM and Keane model is caused by the difference of the EoS function. In other words, there is the EoS function dependence. Here we accept the Keane model as a best fit model.

Table S4. $MgSiO_3$ post-perovskite EoS parameters for the *ab initio* data.

	Room temperature EoS parameters			High Temperature EoS parameters					
Formula	V_0 [Å^3/cell]	K_0 [GPa]	K'_0	γ_0	γ_∞	β	θ_0 [K]	K'_∞	memo
3BM	162.48(5)	242.6(3)	3.980(1)	1.643(6)	0.989(5)	5.07(10)	1102(25)	-	Ab initio, LDA
Vinet	165.74(8)	196.1(6)	4.819(5)	1.464(9)	0.855(41)	1.33(16)	1570(5)	-	Ab initio, LDA
AP2	164.33(5)	214.9(5)	4.482(6)	1.542(6)	0.989(7)	2.80(10)	1471(5)	-	Ab initio, LDA
Keane	164.22(6)	205.4(7)	5.069(19)	1.495(5)	0.818(12)	1.97(7)	995(17)	2.627(2)	Ab initio, LDA, fit8

The numbers in parentheses are uncertainties in the last digit.

References in Supplementary Information

31. Birch, F. Finite elastic strain of cubic crystals. *Phys. Rev.* **71**, 809-824 (1947).
32. Vinet, P., Ferrante, J., Rose, J. H., & Smith, J. R. Compressibility of Solids. *J. Geophys. Res.* **92**, 9319-9325 (1987).
33. Stacey, F.D. The K-primed approach to high-pressure equations of state. *Geophys. J. Int.* **143**, 621-628 (2000).
34. Dorogokupets, P. I., & Oganov, A. R. Ruby, metals, and MgO as alternative pressure scales: a semiempirical description of shockwave, ultrasonic, X-ray, and thermochemical data at high temperatures and pressures. *Phys. Rev. B* **75**, 024115; DOI:10.1103/PhysRevB.75.024115 (2007).
35. Slater, J. C. Introduction to Chemical Physics. New York: McGraw-Hill (1939).
36. Singh, A. K., & Takemura, K. Measurement and analysis of nonhydrostatic lattice strain component in niobium to 145 GPa under various fluid pressure-transmitting media. *J. Appl. Phys.* **90**, 3269-3275; DOI:10.1063/1.1397283 (2001).
37. Nishio-Hamene, D., & Yagi, T. Equations of state for postperovskite phases in the $MgSiO_3$–$FeSiO_3$–$FeAlO_3$ system. *Phys. Earth Planet. Inter.* **175**, 145-150; DOI:10.1016/j.pepi.2009.03.006 (2009).
38. Tange, Y., Kuwayama, Y., Irifune, T., Funakoshi, K., & Ohishi, Y. P-V-T equation of state of $MgSiO_3$ perovskite based on the MgO pressure scale: a comprehensive reference for mineralogy of the lower mantle. *J. Geophys. Res.* **117**, B06201; DOI:10.1029/2011JB008988 (2012).
39. Ohishi, Y., Hirao, N., Sata, N., Hirose, K., & Takata, M. Highly intense monochromatic X-ray diffraction facility for high-pressure research at SPring-8. *High Press. Res.* **28**, 163–173; DOI:10.1080/08957950802208910 (2008).

40. Hohenberg P., & Kohn, W. Inhomogeneous Electron Gas. *Phys. Rev.* **136**, B864; DOI:10.1103/PhysRev.136.B864 (1964).

41. Kohn, W., & Sham, L. J. Self-consistent equations including exchange and correlation effects. *Phys. Rev.* **140**, A1133; DOI:10.1103/PhysRev.140.A1133 (1965).

42. Ceperley, D. M. & Alder, B. J. Ground State of the Electron Gas by a Stochastic Method. *Phys. Rev. Lett.* **45**, 566; DOI:10.1103/PhysRevLett.45.566 (1980).

43. Perdew, J. P., & Zunger, A. Self-interaction correction to density-functional approximations for many-electron systems. *Phys. Rev. B* **23**, 5048; DOI: 10.1103/PhysRevB.23.5048 (1981).

44. Dekura, H., Tsuchiya, T., & Tsuchiya, J. Ab initio lattice thermal conductivity of $MgSiO_3$ perovskite as found in Earth's lower mantle. *Phys. Rev. Lett.* **110**, 025904; DOI:10.1103/PhysRevLett.110.025904 (2013).

45. Troullier. N., & Martins, J. L. Efficient pseudopotentials for plane-wave calculations. *Phys. Rev. B* **43**, 1993; DOI:10.1103/PhysRevB.43.1993 (1991).

46. Giannozzi, P. *et al.* QUANTUM ESPRESSO: a modular and open-source software project for quantum simulations of materials. *J. Phys.: Condens. Matter.* **21**, 395502; DOI:10.1088/0953-8984/21/39/395502 (2009).

47. Monkhorst, H. J., & Pack, J. D. Special points for Brillouin-zone integrations. *Phys. Rev. B* **13**, 5188; DOI:10.1103/PhysRevB.13.5188 (1976).

48. Baroni, S., de Gironcoli, S., Corso, A. D., & Giannozzi, P. Phonons and related crystal properties from density-functional perturbation theory. *Rev. Mod. Phys.* **73**, 515; DOI:10.1103/RevModPhys.73.515 (2001).

49. Blöch, P. E., Jepsen, O., & Andersen, O. K. Improved tetrahedron method for Brillouin-zone integrations. *Phys. Rev. B* **49**, 16223; DOI:10.1103/PhysRevB.49.16223 (1994).

50. Baroni, S., Giannozzi, P., & Isaev, E. Density-functional perturbation theory for quasi-harmonic calculations. *Rev. Mineral. Geochem.* **71**, 39; DOI:10.2138/rmg.2010.71.3 (2010).

51. Wu, Z. Calculating the anharmonic free energy from first principles. *Phys. Rev. B* **81**, 172301; DOI:10.1103/PhysRevB.81.172301 (2010).

52. Zhang, D., Sun, T., Wentzcovitch, R. M. Phonon Quasiparticles and Anharmonic Free Energy in Complex Systems. *Phys. Rev. Lett.* **112**, 058501; DOI:10.1103/PhysRevLett.112.05850 (2014).

53. Grabowski, B., Ismer, L., Hickel, T., & Neugebauer, J. Ab initio up to the melting point: Anharmonicity and vacancies in aluminum. *Phys. Rev. B* **79**, 134106; DOI:10.1103/PhysRevB.79.134106 (2009).

54. Stoer, J., & Bulirsch, R. Introduction to numerical analysis (Springer-Verlag New York Inc) (1980).
